# Sleep behaviors and metabolic-associated fatty liver disease

**DOI:** 10.1371/journal.pone.0323715

**Published:** 2025-05-28

**Authors:** Yuqing Cai, Jia Chen, Xiaoyu Deng, Caiyun Wang, Jiefeng Huang, Ningfang Lian

**Affiliations:** 1 Department of respiratory and critical care medicine, Respiratory Disease Research Institute, the First Affiliated Hospital, Fujian Medical University, Fuzhou, China; 2 Department of respiratory and critical care medicine, National Regional Medical Center, Binhai Campus of the First Affiliated Hospital, Fujian Medical University, Fuzhou, China; Universita degli Studi della Campania Luigi Vanvitelli Scuola di Medicina e Chirurgia, ITALY

## Abstract

**Objective:**

This study aimed to comprehensively evaluate the association between various sleep behaviors and the risk of metabolic-associated fatty liver disease (MAFLD), particularly self-reported snoring.

**Methods:**

Multivariate logistic regression was used to explore independent factors associated with MAFLD. ROC curve and decision curve analyses were used to analyze and compare the different indicators.

**Results:**

A total of 3708 patients were enrolled, and 41.4% of them had MAFLD. According per multivariate logistic regression analysis, self-reported snoring was an independent predictor of MAFLD (*p* < 0.001), particularly the occasional and frequent snoring groups [OR (95% CI): 1.44 (1.12–1.87), 1.48 (1.15–1.91), *p* < 0.001]. In addition, the liver function levels and incidence rates of metabolic parameters were independently associated with the severity of self-reported snoring (all *p* < 0.05). Subgroup analyses suggested that the frequency of snoring was independently related to the risk of MAFLD in young and middle-aged patients (both *p* < 0.05), and was no longer associated with any frequency of self-reported snoring in the subgroup older than 60 years (*p* = 0.400). In both female and male subgroup, subjects who snored frequently had a higher odds risk of MAFLD than those who did not (both *p* < 0.05). The area under the ROC curve for snoring was 0.638, which was superior to that of the other indicators for MAFLD prediction (all *p* < 0.001). Meanwhile, decision curve analysis showed that snoring had a better clinical net benefit compared to other biomarkers, with a threshold probability (Pt) of approximately 0.3–0.6.

**Conclusion:**

Self-reported snoring was an independent risk factor for MAFLD in young and middle-aged subjects with a moderate predictive value. Therefore, intense monitoring and evaluation of MAFLD in these patients is necessary.

## Introduction

Metabolic-associated fatty liver disease (MAFLD) is a common chronic liver disease. The global prevalence of MAFLD has increased from 25% in 2016 to 32% by 2022 [1]. MAFLD is defined as a systemic metabolic disorder involving the liver, that is, hepatic steatosis combined with overweight, type 2 diabetes, or metabolic dysfunction [2]. The pathophysiological mechanisms underlying MAFLD have not yet been fully elucidated. Insulin resistance (IR), oxidative stress, endoplasmic reticulum stress, mitochondrial dysfunction, and other factors jointly act on genetically susceptible patients, inducing lipid accumulation in hepatocytes and leading to fatty liver [3]. Exploring the risk factors for MAFLD is currently a hot research topic.

Sleep is a fundamental physiological requirement of human health. Subjects with increased upper airway resistance often suffer from snore[4]. Snoring is highly prevalent among this population [5]. Previous research has found that snoring plays an important role in various metabolic diseases such as diabetes, hypertension, and kidney disease [6–8]. Accumulated evidences suggested that obstructive sleep apnea (OSA) is a risk factor of NAFLD, even in non-obese people [9,10]. The correlation between snoring and MAFLD, one of the common symptoms of OSA, has received little attention. In addition, as we know, NAFLD and type 2 diabetes mellitus (T2DM) often coexist and work together to increase the risk of adverse clinical outcomes [11]. While other sleep patterns, such as sleep duration, daytime sleepy and insomnia were independently associated with incident type 2 diabetes [12,13].

Thus, the purpose of the present study was to further explore the association between various sleep behaviors and the risk of MAFLD and to provide a reference for the early screening of MAFLD.

## Materials and methodss

### Study population

The data in this study were derived from the NHANES (National Health and Nutrition Examination Surveys) 2017–2018. The NHANES evaluates the health status and nutritional status of the USA population over a repeated 2-year cycle. All procedures performed in this study involving human participants were in accordance with the ethical standards of the institutional and/or national research committee and with the 1964 Helsinki Declaration and its later amendments or comparable ethical standards. Detailed information on the ethics application and written informed consent were provided by the NHANES website (https://www.cdc.gov/nchs/nhanes/about/erb.html).

**Fig 1:**
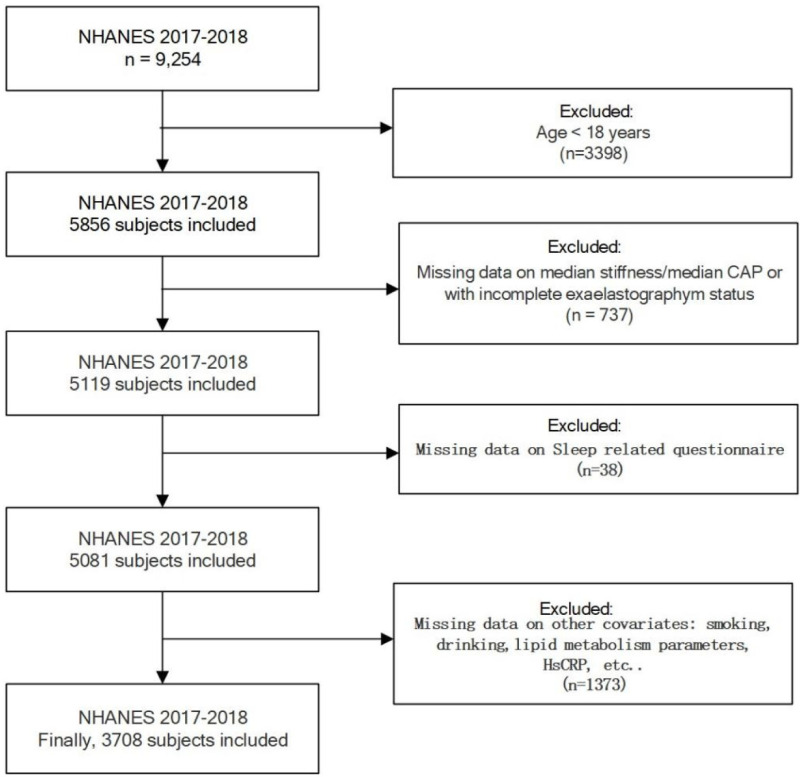
Flow chart of study population enrollment.

The demographics, race, disease history, results of FibroScan, blood biochemical tests, and sleep-related data were screened from the NHANES 2017–2018.Eligible participants were over 18 years old. We excluded participants with incomplete information on FibroScan (n = 737), sleep related questionnaire(n = 38) and other covariates (smoking, drinking, lipid metabolism parameters, etc.; n = 1373). A total of 3708 participants were thus eligible for inclusion (Fig 1).

### Definition of hepatic steatosis, liver fibrosis and MAFLD

Hepatic steatosis and fibrosis were diagnosed by transient elastography (FibroScan). Hepatic steatosis was defined as the values of the controlled attenuation parameter (CAP) greater than 274. Liver fibrosis was defined as the thresholds of liver stiffness measurements (LSM) greater than 9.7 [14]. Examinations were considered reliable only if ≥10 LSMs were obtained after a fasting period of ≥3 h with an interquartile range/median of < 30%.

MAFLD was diagnosed based on confirmed hepatic steatosis and at least one of the following conditions[2]: BMI > 25 kg/m2, diabetes mellitus, or at least two metabolic risk factors. The metabolic risk factors included the following indicators: 1) waist circumference ≥ 102 cm in male or ≥ 88 cm in female; 2) arterial blood pressure ≥130/85 mmHg or with anti-hypertension treatments; 3) triglyceride (TG) ≥1.70 mmol/L or with lipid-lowering treatment; 4) high-density lipoprotein cholesterol (HDL-C) <1.0 mmol/L in male or HDL-C < 1.3 mmol/L for female; 5) prediabetes; 6) hypersensitive C-reactive protein >2 mg/L.

### Definition of sleep disorder and other covariates

Information on sleep behavior was obtained using a questionnaire. Participants were asked, “How often do you snore?” or “How often do you snort or stop breathing?” “Never” was defined without the self-reported snoring, or snort/stop breathing. “Rarely” was defined 1–2 nights a week. “Occasionally” was defined 3–4 nights a week. “Frequently” was defined 5 or more nights a week. How often do you feel overly sleepy during the day? “Never” was defined without sleepy. “Rarely” was defined 1 time a month. “Sometimes” was defined 2–4 times a month. “Often” was defined 5–15 times a month. Those “Refused” or “Do not know” were excluded from the study.

Sleep duration was calculated from self-reported sleep durations on weekdays and workdays. According to the recommendations of the American Academy of Sleep Medicine and Sleep Research Society, 7–9 hours of sleep is recommended [15]. Sleep duration was categorized as < 7, 7–9, or ≥ 9 hours.

The covariates were selected based on substantive reasoning and previous studies. Race was categorized as Mexican American, other Hispanics, non-Hispanic white, non-Hispanic black, and others.

### Statistical analysis

Continuous variables are expressed as the mean ± standard deviation. The Kolmogorov–Smirnov test was used to examine the normality of the continuous variables. The mean imputation technique is utilized to fill in missing values. Differences between non-normally distributed continuous variables were analyzed using the Mann–Whitney U-test, while normally distributed variables were analyzed using Student’s t-test. Categorical variables are expressed as percentages and analyzed using the chi-square test. Multivariate logistic regression was used to explore independent factors associated with MAFLD. ROC curve and decision curve analyses were used to analyze and compare the different indicators. Statistical significance was set at *p* < 0.05. All analyses were conducted the R 3.6.2 (https://www.r-project.org/).

### Patient and public involvement

Patients and the public were not involved in the design, or conduct, or reporting, or dissemination plans of this research.

## Results

### The basic characteristics and sleep parameters of participants

In total, 9254 individuals were included in the interviews. After excluding participants with missing data on sleep-related questionnaires, median stiffness/median CAP, other covariates, incomplete elastography exam status, and age < 18 years, 3708 subjects were included in the final analysis.

The recruited subjects were 1912 male and 1796 female. The mean age of the patients was (49 ± 18) years. 1534 (41.4%) patients met the diagnostic criteria for MAFLD. Subjects with MAFLD were more likely to be male, with a high risk of liver fibrosis, self-reported snoring, self-reported snoring, stopped breathing, and shorter sleep time. Regarding biochemical features, the MAFLD group had higher levels of ALT, AST, GGT, LDH and Uric acid than the control group (all *p* < 0.05). The Ethnic composition ratio differed between patients with and without liver injury (*p* < 0.05). The baseline characteristics of all the subjects are summarized in Table 1.

**Table 1:** The Basic Characteristics and Sleep Parameters of Patients with or without MAFLD.

	Overall	Non-MAFLD	MAFLD	p-value
**N**	3,708	2174	1534	
**Age (y)**	49 ± 18	47 ± 19	53 ± 16	<0.001
**Gender (male) n (%)**	1,912 (52)	1,011 (47)	901 (59)	<0.001
**Race n (%)**				<0.001
Mexican American	533 (14)	243 (11)	290 (19)	
Other Hispanic	350 (9.4)	198 (9.1)	152 (9.9)	
Non-Hispanic White	1,360 (37)	795 (37)	565 (37)	
Non-Hispanic Black	837 (23)	550 (25)	287 (19)	
Other/multiracial	628 (17)	388 (18)	240 (16)	
**BMI (kg/m**^**2**^)	30 ± 7	27 ± 6	34 ± 7	<0.001
**Drinking n (%)**	1,837 (50)	1,124 (52)	713 (46)	0.002
**Smoking status n (%)**				<0.001
Current smoker	709 (19)	447 (21)	262 (17)	
Former smoker	931 (25)	463 (21)	468 (31)	
Never smoker	2,068 (56)	1,264 (58)	804 (52)	
**Diabetes n (%)**	726 (20)	239 (11)	487 (32)	<0.001
**Hypertension n (%)**	1,885 (53)	890 (42)	995 (67)	<0.001
**Clinical measures**				
**Waist circums (cm)**	101 ± 17	93 ± 14	112 ± 15	<0.001
**Hip circums (cm)**	107 ± 15	102 ± 12	114 ± 15	<0.001
**ALT (U/L)**	23 ± 18	20 ± 16	27 ± 19	<0.001
**AST (U/L)**	22 ± 13	21 ± 12	23 ± 13	<0.001
**ALP** (U/L)	78 ± 27	76 ± 28	82 ± 25	<0.001
**Glucose** (mmol/l)	5.64 ± 1.91	5.29 ± 1.52	6.13 ± 2.26	<0.001
**γ-GT** (U/L)	32 ± 44	27 ± 43	40 ± 46	<0.001
**LDH** (U/L)	157 ± 34	156 ± 34	160 ± 35	<0.001
**Cholesterol** (mmol/l)	4.84 ± 1.07	4.79 ± 1.04	4.92 ± 1.10	<0.001
**Triglyceride** (mmol/l)	1.63 ± 1.32	1.33 ± 1.09	2.04 ± 1.50	<0.001
**HsCRP (mg/l)**	4.0 ± 7.6	3.1 ± 6.4	5.2 ± 8.8	<0.001
**Insulin (uU/mL)**	14 ± 19	10 ± 11	21 ± 26	<0.001
**Sleep behaviors**				
**Sleeptime n (%)**				0.034
7-9 h	2,051 (55)	1,198 (55)	853 (56)	
<7 h	918 (25)	515 (24)	403 (26)	
>9 h	739 (20)	461 (21)	278 (18)	
**Snort or stop breathing n (%)**				<0.001
Never	2,651 (71)	1,660 (76)	991 (65)	
Rarely	490 (13)	265 (12)	225 (15)	
Occasionally	248 (6.7)	110 (5.1)	138 (9.0)	
Frequently	194 (5.2)	73 (3.4)	121 (7.9)	
**Excessive daytime sleepiness n (%)**				<0.001
Never	533 (14)	329 (15)	204 (13)	
Rarely	893 (24)	530 (24)	363 (24)	
Sometimes	174 (4.7)	76 (3.5)	98 (6.4)	
Often	151 (4.1)	58 (2.7)	93 (6.1)	
**Self-reported Snoring n (%)**				<0.001
Never	916 (25)	677 (31)	239 (16)	
Rarely	911 (25)	598 (28)	313 (20)	
Occasionally	763 (21)	416 (19)	347 (23)	
Frequently	1,118 (30)	483 (22)	635 (41)	
**Self-reported trouble sleeping n (%) nmn,(%) n,(%)n,(%) (n, %)**	1,067 (29)	554 (25)	513 (33)	<0.001
**Liver fibrosis n (%)**	272 (7.3)	66 (3.0)	206 (13)	<0.001

Abbreviations: BMI: body mass index, ALT: alanine transaminase, AST: aspartate transaminase, ALP: alkaline phosphatase, GGT: gamma-glutamyl transferase, LDH: lactate dehydrogenase, HsCRP: plasma high sensitivity C-reactive protein.

### The associations between sleep factors and MAFLD

A multivariate regression analysis was performed to evaluate the risk of sleep factors associated with MAFLD. Odds ratios (OR) were calculated for three models. Model 1 was adjusted for sleep factors including sleep duration, self-reported snoring, snorting or stopping breathing, daytime sleepiness, and self-reported sleep disorders. Model 2 was adjusted for the aforementioned factors, age, sex, and race. Model 3 was adjusted for the factors in model 2 plus the presence of diabetes and hypertension, BMI, history of smoking and drinking, triglyceride, uric acid, and hsCRP.

The results of the multivariate regression analysis (Table 2) showed that MAFLD was independently associated with different severities of self-reported snoring in both models 1 and 2 (both *p* < 0.05). The self-reported snoring group with higher severity had higher OR values. After adjusting for sleep and anthropometric factors, frequent self-reported snoring was independently associated with MAFLD (ORs were around 3 in both models 1 and 2, *p* < 0.01). After additionally adjusting for metabolic-related factors, occasional and frequent snoring was still independently associated with MAFLD, while the association between rare snoring and MAFLD was attenuated and was no longer statistically significant (Model 3, OR=1.03, 95% CI: 0.80–1.33). Sleep duration (>9 h) and self-reported sleep disorders were independently associated with MAFLD when only adjusted for sleep and anthropometric factors (Model 2, OR=1.554, 95% CI: 1.219–1.982 and OR = 1.34, 95% CI:1.14–1.57, respectively; *p* < 0.001). However, after adjusting for metabolism-related factors, this association was not statistically significant (Model 3, OR=0.8, 95% CI: 0.64–0.10, *p* = 0.10).

**Table 2:** The associations between sleep factors and MAFLD.

		Model 1			Model 2			Model 3	
	OR	95% CI	P value	OR	(95% CI)	P value	OR	(95% CI)	P value
**Self-reported Snoring**			<0.001			<0.001			<0.001
Never	Reference	—		Reference	—		Reference	—	
Rarely	1.45	1.18, 1.78		1.40	1.13, 1.73		1.03	0.80, 1.33	
Occasionally	2.25	1.82, 2.77		2.06	1.66, 2.56		1.44	1.12, 1.87	
Frequently	3.33	2.72, 4.08		2.91	2.37, 3.59		1.48	1.15, 1.91	
**Excessive daytime sleepiness**			>0.9			0.7			0.5
Never	Reference			Reference	—		Reference	—	
Rarely	1.06	0.85, 1.34		1.11	0.88, 1.41		1.04	0.79, 1.37	
Sometimes	1.02	0.57, 1.82		1.21	0.67, 2.18		1.25	0.62, 2.56	
Often	0.82	0.39, 1.69		0.86	0.40, 1.80		0.49	0.20, 1.21	
**Sleep duration**			0.085			0.035			0.10
7-9 h	Reference	—		Reference	—		Reference	—	
< 7 h	1.02	0.86, 1.20		1.06	0.90, 1.26		1.02	0.83, 1.25	
> 9 h	0.83	0.69, 0.99		0.81	0.68, 0.98		0.80	0.64, 1.00	
**Self-reported trouble sleeping**			<0.001			<0.001			0.5
No	Reference	—		Reference	—		Reference	—	
Yes	1.35	1.15, 1.57		1.34	1.14, 1.57		1.02	0.84, 1.23	
**Snort or stop breathing**			0.3			0.3			0.4
Never	Reference	—		Reference	—		Reference	—	
Rarely	1.14	0.93, 1.40		1.11	0.90, 1.36		0.94	0.74, 1.21	
Occasionally	1.29	0.79, 2.11		1.20	0.73, 1.98		0.89	0.49, 1.63	
Frequently	1.70	0.90, 3.36		1.75	0.91, 3.47		1.92	0.86, 4.34	

### The characteristics of participants based on Self-reported snoring frequency

We compared the liver function and metabolic parameters in subjects with different severities of self-reported snoring (Table 3). BMI, waist circumference, hip circumference, glucose, cholesterol, triglyceride, uric acid, serum insulin, ALT, and AST levels increased with increased snoring frequency (all *p* < 0.001). The incidence rates of hypertension, diabetes, MAFLD, and liver fibrosis increased with increased snoring frequency (all *p* < 0.001).

**Table 3:** The characteristics of participants based on Self-reported snoring frequency.

Self-reported snoring	Never	Rarely	Occasionally	Frequently	p-value
**N**	916	911	763	1118	
**BMI (kg/m**^**2**^)	27 ± 6	29 ± 7	30 ± 7	32 ± 8	<0.001
**Waist circums (cm)**	93 ± 17	98 ± 16	102 ± 16	108 ± 17	<0.001
**Hip circums (cm)**	103 ± 13	106 ± 13	108 ± 14	111 ± 15	<0.001
**ALT (U/L)**	21 ± 21	23 ± 17	23 ± 17	25 ± 16	<0.001
**AST (U/L)**	22 ± 15	22 ± 12	21 ± 12	23 ± 12	0.002
**Glucose** (mmol/l)	5.44 ± 1.57	5.52 (1.75)	5.62 (1.82)	5.90 (2.28)	<0.001
**cholesterol**(mmol/l)	4.74 ± 1.06	4.85 (1.07)	4.84 (1.00)	4.93 (1.10)	<0.001
**triglyceride**(mmol/l)	1.42 ± 1.16	1.57 (1.16)	1.59 (0.93)	1.87 (1.71)	<0.001
**HsCRP (mg/l)**	3.6 ± 7.8	3.6 ± 6.7	4.4 ± 8.0	4.4 ± 7.7	<0.001
**Insulin (uU/mL)**	11 ± 11	13 ± 26	15 ± 17	17 ± 20	<0.001
**Diabetes n (%)**	147 (16)	127 (14)	155 (20)	297 (27)	<0.001
**Hypertension n (%)**	365 (41)	410 (47)	413 (56)	697 (65)	<0.001
**MAFLD n (%)**	239 (26)	313 (34)	347 (45)	635 (57)	<0.001
**Liver fibrosis n (%)**	60 (6.6)	51 (5.6)	50 (6.6)	111 (9.9)	<0.001

Abbreviations: BMI: body mass index, ALT: alanine transaminase, AST: aspartate transaminase, HsCRP: plasma high sensitivity C-reactive protein, MAFLD: Metabolic-associated fat liver disease.

### Subgroup analysis

**Fig 2:**
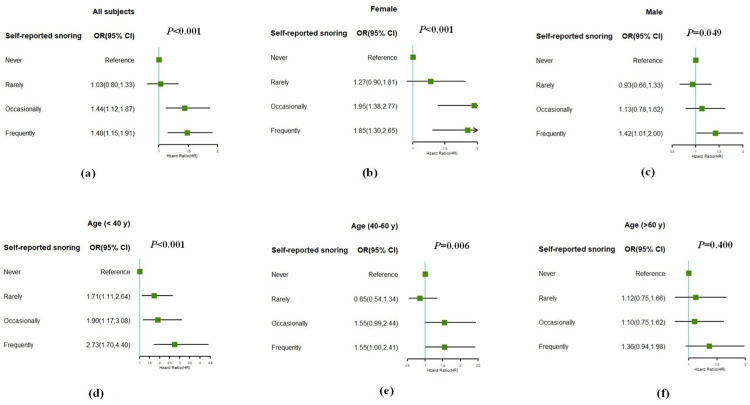
Subgroup analyses stratified by gender and age. (a) in all subjects, (b) subgroup analyses of female, (c) subgroup analyses of male, (d) subgroup analyses of subjects under 40 years, (e) subgroup analyses of subjects aged 40–60 years, (f) subgroup analyses of subjects over 60 years.

To determine whether self-reported snoring was associated with MAFLD in different age and sex subgroups, subgroup analysis was performed. As shown in Fig 2, both in the female and male subgroup, subjects who snored frequently had a higher odds risk of MAFLD than those who did not (both *p* < 0.05). The frequency of snoring was independently associated with MAFLD in the subgroups aged < 40y and 40-60y. while the 40-60y subgroup had lower OR. MAFLD was no longer associated with any frequency of self-reported snoring in the subgroup aged > 60 years (*p* = 0.400). Self-reported snoring was an independent risk factor of MAFLD in young and middle-aged subjects.

**Fig 3:**
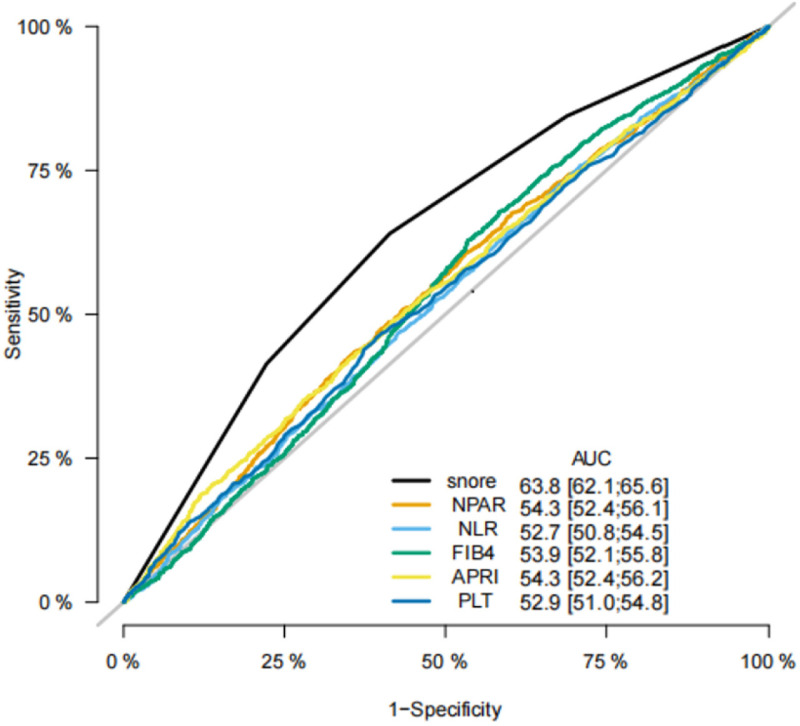
The ROC curve of snoring, NPAR, NLR, FIB4, APRI and PLT.

**Fig 4:**
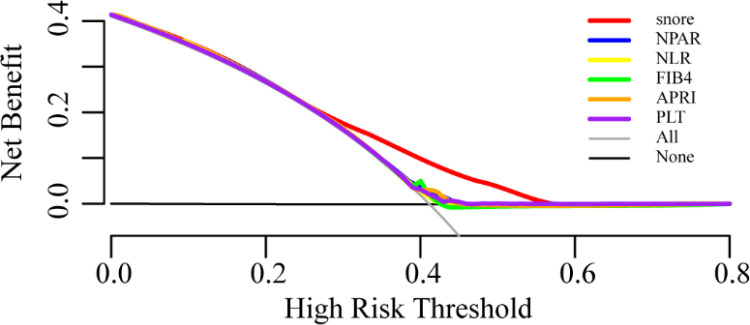
The decision curve analysis of snoring, NPAR, NLR, FIB4, APRI and PLT.

In addition, comparing with other biomarkers for MAFLD, including fibrosis-4 (FIB-4), platelet (PLT), aspartate aminotransferase to platelet ratio index (APRI), the neutrophil-to-albumin ratio (NPAR) and the neutrophil-to-lymphocyte ratio (NLR), the area under ROC curve of snoring was 0.638, which is superior to the other indicators mentioned above and has statistical differences (Fig 3, all *p* < 0.001). Meanwhile, decision curve analysis showed that snoring had a better net clinical benefit than other biomarkers, with a threshold probability (Pt) of approximately 0.3–0.6 (Fig 4).

## Discussion

This study showed that self-reported snoring was an independent risk factor for MAFLD in young and middle-aged subjects with a moderate predictive value. This association was not affected by sex, race, or other metabolic factors such as diabetes, hypertension, and BMI. Subjects with frequent snoring had higher OR. Liver function levels and incidence rates of metabolic parameters were independently associated with the severity of self-reported snoring. Compared with FIB-4, APRI, NPAR, and NLR, snoring had a better ROC curve area and net clinical benefit in MAFLD prediction.

In this study, the associations assessed between the self-reported snoring and MAFLD were in line with the results of the previous research [16,17]. The difference is that compared to the study by Wang [16], we included more sleep behaviors based on the same population. Sleep behaviors typically interact with each other and affect the development of various diseases [18]. According to a cross-sectional study conducted in China, multiple sleep behaviors are associated with MAFLD risk [17]. Including more sleep behaviors is clearly appropriate and reliable. After excluding confounding factors, self-reported snoring was confirmed as an independent risk factor. Apart from self-reported snoring, there were no statistically significant differences in sleep duration, snort or stop breathing, daytime sleepiness, and self-reported sleep disorders between the MAFLD and non-MAFLD groups after multiple logistic regression, which was inconsistent with some previous studies [17,19]. These differences may be related to the different participants included in previous studies.

In addition, the diagnosis of hepatic steatosis and liver fibrosis was based on the FibroScan. Previous studies have shown that CAP and LSM using FibroScan are accurate noninvasive methods for assessing liver steatosis and fibrosis in patients with NAFLD [20]. It is predicted to eliminate the need for biopsy in at least 45.1% of patients [21]. Compared to invasive liver biopsy, noninvasive methods are clearly more suitable for clinical screening [22]. FibroScan is the most widely used because it yields the best tradeoff between accurate results and simple operations [23].

Moreover, in the subgroup analysis, the positive association was stronger in females than in males, which may be related to different hormone levels. There were statistically significant differences between the young and middle-aged participants. This finding is consistent with previous studies based on the same population [16]. This may be attributed to the stronger oxidative stress response in younger participants. Nevertheless, a study from China showed the predictive value of snoring in middle-aged and elderly population [17]. Further research is required to elucidate the mechanisms that underlie these differences. Additionally, indicators such as FIB-4, APRI, NPAR, and NLR, which can reflect the inflammatory status, are mostly used to predict MAFLD [24–26]. In this study, the predictive value of snoring was optimal, differing from previous studies [27]. The predictive value of other indicators may be limited by the characteristics of the included population and the sample size. It is worth mentioning that a study by Liu [26], based on the same population, showed that the AUROC of NPAR was 0.810 (95% CI: 0.794–0.825) for NAFLD in nondiabetic individuals. In this study, NPAR had a low predictive value and was unaffected by diabetes. The difference may be caused by the different settings of the CAP cutoff points, which have different sensitivities. The characteristics of the included population may affect the predictive value of the NPAR [20].

Although snoring has only a moderate predictive value as a non-invasive and simple indicator, it provides a valuable starting point for initial disease screening and serves as a reference for constructing more accurate models in the future. For example, in outpatient settings, enhancing MAFLD screening and follow-up for the aforementioned patients may optimize resource allocation and improve clinical efficiency. This study has some limitations. First, the retrospective nature of this study may have compromised this conclusion. Future prospective studies are essential to further establish causal relationships. Second, subjective data such as snoring may have affected the results. Finally, due to data limitations, we could not incorporate additional sleep behaviors, such as ease of getting up in the morning and chronotypes. In future research, it is also necessary to establish a global platform to further clarify the relationship between snoring and MAFLD.

## Conclusion

Overall, self-reported snoring, especially occasional and frequent snoring, was an independent risk factor of MAFLD in young and middle-aged participants. Therefore, intense monitoring and evaluation of MAFLD in these patients is necessary.
